# The past and future of “sex genes”

**DOI:** 10.1515/medgen-2023-2040

**Published:** 2023-08-16

**Authors:** Christoph Rehmann-Sutter, Nadine Hornig, Birgit Stammberger, Heiko Stoff

**Affiliations:** Universität zu Lübeck Institut für Medizingeschichte und Wissenschaftsforschung Königstraße 42 23552 Lübeck Deutschland; Christian-Albrechts-Universität zu Kiel Institut für Humangenetik Schwanenweg 24 24105 Kiel Deutschland; Universität zu Lübeck Institut für Medizingeschichte und Wissenschaftsforschung Königstraße 42 23552 Lübeck Deutschland; Medizinische Hochschule Hannover Institut für Ethik, Geschichte und Philosophie der Medizin Carl-Neuberg-Str. 1 30625 Hannover Deutschland

**Keywords:** sex genes, sex determination, history of hormone research, history of genetics, feminist feminist science studies

## Abstract

Much later than the discovery of “sex chromosomes” and of “sex hormones”, genetics started delivering detailed explanations of sex-determining developmental pathways. Despite increasing knowledge of biological processes, concepts and theories about sex development are never based on facts alone. There are inevitable entanglements of biological description and changing cultural assumptions and they play a key role in how sex genes are framed and interpreted in biological research. In this review article we first focus on the early 20th century biology that worked in a hormone-based paradigm. Genetic explanations emerged later, first on the basis of sex chromosomes; starting in the 1980s, on the basis of genes. We highlight orthodox views of female development, which saw the default pathway of human sex development. We will show how recent findings in biology challenge it. The article discusses the interactions of causal claims in science with cultural assumption about gender and outlines three influential strands of critical feminist philosophy of science: the critique of genetic determinism and genetic essentialism, of dualist assumptions, and of an androcentric bias in the conception of research strategies. In the final section we suggest key agenda points of future genetic research on sex determination.

Sex chromosomes were first identified in 1905, but it was only much later that genetics could actually provide detailed descriptions of sex-determining developmental pathways. Early 20th-century biology and biological studies of sex determination primarily used a hormone-based research paradigm that gave ample opportunities for experimental interventions and was closely linked to 19th-century physiological research – primarily elucidating the features of the female body through its differences to the male.

## 1 The entry of genetics into early 20th-century research on sex dimorphism

According to a much-discussed thesis of historian Thomas Laqueur, the ‘two sex model’ was invented by anatomical and physiological science in the 18th century. Before that, man and woman had been understood in the Western world as specific manifestations of one single sex. With the shift to the ‘two sex model’ the focus of modern medical research was the female body which has been interpreted as the somatic ‘other’ of the male-human body [1]. The socio-historical polarisation of gender characteristics created an order of biological difference that identified women and men on the basis of binary oppositions and fixed them as psychophysical opposites. Nevertheless, scientists had resigned themselves to the fact that this order never really and comprehensively worked: it simply produced too many exceptions. Radical incomparability, which was so defining for the 19th century, fell into a serious crisis among other factors due to scientific research.

In his 1933 lecture on “Femininity” the founder of psychoanalysis Sigmund Freud recapitulated this discussion from a view of psychosexual theory by stating that the everyday certainty of the distinction between “male” and “female” is shared scientifically at only one point: in mammals, male is characterised by the possession of spermatozoa, female by possession of the egg. But biological research has proven that the presumed fundamental difference between male and female is not stable and parts of the male sexual apparatus are also found in the body of the female and vice versa. The individual is just not man or woman, so Freud concluded, but always both, in an unequal admixture and subject to considerable variation. He thus concluded that what constitutes masculinity or femininity is an unknown characteristic not captured by anatomy. Freud participated in an evolutionary-biological and embryological debate that had been going on since 1900 about what was understood as the necessary ontogenetic bisexuality of all beings. The sexes were seen as a “male-female mosaic” [2, 3]. The biological question of what was masculine and what was feminine took on major social significance in the early 20th century. The life sciences were supposed to provide answers. This was particularly true for sex hormone research.[A16]

The 1920s saw an intense public debate about the “feminisation of the male” and the “masculinisation of the female”. A crisis in the gender order was lamented as “degeneration” and “decline” but was also seen as a sign of modernisation and Americanisation [4]. At the same time, sensational sex change experiments in rodents conducted by physiologist Eugen Steinach at the Biological Experimental Station (Vivarium) in Vienna were reported. Steinach’s aim was to identify the foundational locus of sex determination, to prove the specificity of these sex-determining organs, which he had identified as the hormone-producing tissue of the interstitial cells in the gonad. With his experiments Steinach aimed to prove the potential mutability of sex characteristics, if not of sexes, by the replacement of these organs. His method of “experimental feminisation and masculinisation” in guinea pigs consisted of inducing the growth of female sexual characteristics in castrated males by implanting female gonads in them, and male sexual characteristics in castrated females by implanting male gonads in them. He observed both somatic and behavioural feminisation or masculinisation, which he explained by the activity of the sex hormones produced in the interstitial cells in the gonads. The implanted testis or ovary, in Steinach’s view, inhibited the growth of male or female sex characteristics. He referred to a fundamental antagonism between the two, a “battle of the gonads” [5, 6]. This phrase is reminiscent of Wilhelm Roux’s concept of the “struggle of the parts” in the development of the organism [7]. He claimed to have created an “experimental hermaphrodite” and hypothesised that it would be possible to produce various forms of hermaphroditic animals by surgery [5, 8].

**Figure 1:**
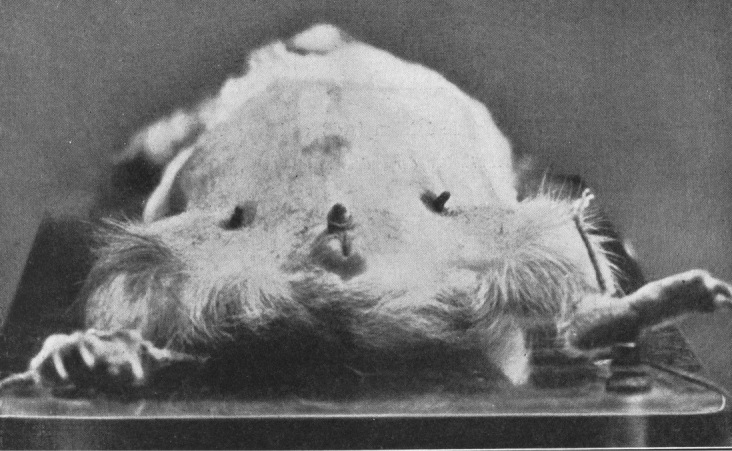
Eugen Steinach’s experimental feminisation of male guinea pigs (from [53]).

Steinach’s experiments seemed to confirm the thesis of the sexologist Magnus Hirschfeld, who understood sex as a mixture of male and female characteristics. Every human being initially carries the potential to be male and female, which, however, only rarely develops into a completely female or male type. Steinach’s experiments enabled this argument to be made in biochemical terms. The so-called theory of intermediate stages was however structured as a binary. It proposed a special status for intersex varieties yet contained an inherent tendency to dissolve the polar gender order; Hirschfeld defined “man” and “woman” as “constructed abstractions” [9]. On the basis of the experimental concept of arbitrarily produced mixed forms, the intermediate stage theory was successively detached from dimorphism in the 1920s. According to the “law of unlimited multiplicity of the sexual constitution”, Hirschfeld stated, there were only transitions, a “gapless series of individual cases” [10]. A biological model of the mixed forms was tied to the development of endocrinological research, which in turn required the chemical isolation of the hormones. As historian of science Nelly Oudshoorn has noted, it was only after the sex hormones had been isolated in pure form around 1930 that they could also be interpreted as chemical substances producing various synergistic effects in the body [11]. They were no longer absolutely, but only relatively sex-specific – which did not change the fact that in the further course of the 20th century evolutionary-biological, psycho-endocrinological or neuroscientific research work stabilised the antagonistic model of the sexes. In 1934 biochemist Adolf Butenandt summarised that chemical research had shown that the three sex-specific gonadal hormones (estradiol, progesterone and testosterone) had close chemical relationships to each other and to the sterols [12]. The organism produces sex hormones by degrading sterols. Steroid research enabled new possibilities for the production of biologically active substances. Dehydroandrosterone, made accessible by oxidative removal of the side chain of cholesterol, served as a technically easily accessible basic substance for the production of all steroid hormones.

Until the 1940s, endocrinology played a decisive role in experimental studies of sex development, while chromosomal theories of sex determination at the moment of fertilisation, developed in the early 1900s, were difficult to prove. Even Richard Goldschmidt’s genetic research on sex determination was deeply influenced by hormonal experiments of the 1910s. Physiologist Alexander Lipschütz assumed an original asexual state of the mammal embryo, with the sexes being promoted or inhibited by the hormones produced in the interstitial cells [13]. Neither the secondary somatic nor the secondary psychic sex characteristics, he concluded, would be invariably predetermined from the outset. Whereas the primary determination of the sexes according to the chromosomes and Mendelian inheritance was undisputed, most biologists were convinced that the sex of the individual is not unchangeably determined at the moment of conception but depends on chemical relationships and milieu influences [14, 15, 16].

## 2 Sexing developmental pathways

### 2.1 Sex chromosomes

From the beginning of the 20th century onwards research shifted towards genetic explanatory models of sex determination. In 1905 Nettie M. Stevens showed that inheritance of the Y chromosome produced male mealworms [17]. Subsequent experiments in the fruit fly *Drosophila melanogaster* revealed that changes in the ratio of sex chromosomes (gonosomes) and autosomes produced intersex flies, underlining the importance of a specific gonosome number for a specific sex development [18]. In the mid 1940s, A. H. Sturtevant described the transformation of female to male fruit flies through homozygous expression of the autosomal transformer gene *tra*, extending the influence of genetic sex determination outside the sex chromosomes [19]. In 1957 Mathilde Denon and Leo Sachs summarised three sets of causal factors for intersexuality: 1. single gene mutations, 2. abnormal distribution of sex chromosomes, and 3. endocrine dysfunction induced by external factors or by genetic factors affecting the endocrine system, meaning steroid hormone production and action [20]. The advancement of cytological methods made it possible to assess human sex chromosomes [20]. This made it possible to give individuals with a difference in gonosome numbers a molecularily proven diagnosis of difference in sex development (DSD). DSD defines a group of congenital conditions in which chromosomal, gonadal or anatomical sex is atypical. There are currently three main subclasses of DSD: sex chromosome DSDs, 46,XY DSDs, and 46,XX DSDs. Sex chromosome DSD is considered to comprise Turner Syndrome (45,X), Klinefelter Syndrome (47,XXY) and sex chromosome mosaicism (45,X/46,XY and 46,XX/46,XY). 46,XY DSDs and 46,XX DSDs comprise differences of gonadal development, differences of steroid hormone synthesis and differences of hormone action [21].

### 2.2 Genetic sex development

The identification of genes involved in sex development began in the mid-1980s due to advancements in cloning and sequencing technologies. Genetic factors leading to DSD are currently divided into genes involved in gonadal development, in steroid hormone biosynthesis and in steroid hormone action. Mutations in these genes can lead to both 46,XY and 46,XX DSD. Notably, most of these genes are autosomal [22].

**Figure 2:**
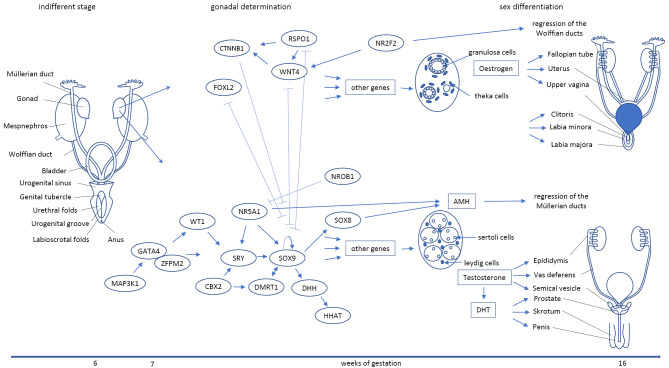
Human sex development from the indifferent gonad to sex differentiation is a concerted interplay of numerous genetic and hormonal factors. Shown are gene products known to be involved in human sex development that are mutated in individuals with DSD. Androgens (Testosterone and Dihydrotestosterone (DHT)) exert their role through the androgen receptor. The role of estrogens in female sex differentiation is poorly understood. Indifferent gonad with undifferentiated genitalia (left). Female (above) and male (below) gonadal determination and sex differentiation. (modified from [29]).

The first mammalian sex determining gene, the sex determining region on the Y chromosome (*SRY*), was described in 1990 [23] and is still considered as a “master switch” that induces male sex development. In humans, 46,XY individuals lacking the *SRY* gene do not develop male gonads and appear phenotypically female at birth. In contrast, a translocation of the *SRY* gene on the X chromosome or autosomes in 46,XX individuals is associated with a male genital development but hypogonadism and infertility, indicating that SRY is necessary but not sufficient for testis development. Indeed, numerous other genes have now been identified as involved in testis development [24] (Figure 2).

Female sex development has long been considered as a default or passive pathway, where the absence of *SRY* in 46,XX individuals was considered to be sufficient to explain ovarian development. Today however, several genes involved in ovarian development have been described [25, 26] although a master switch for ovarian development remains elusive (Figure 2). The observation that 45,X women (Turner syndrome) develop streak gonads (hypoplastic ovaries without steroid hormone production and a persisting tubular structure) suggests a dosage-sensitive factor on the X chromosome that if expressed only from one allele impairs ovarian development. So far only one dosage-sensitive gene on the X chromosome has been described, *NR0B1* (DAX-1), which has been shown to be involved in both ovarian and testicular development. When overexpressed it impedes testis development in 46,XY individuals [27]. No loss of function mutations in *NR0B1* have been described in 46,XX individuals with ovarian dysgenesis so far.

At around 8 weeks post-conception the developing gonads start to express genes involved in steroid hormone biosynthesis. Several pathways leading to the production of androgens as well as estrogens have been identified. Steroid hormone production has been shown to occur in embryonic testis and ovaries; sex-specific hormone production in males peaks from 10 to 15 wpc for androgens and in a similar time window in females for estrogens [28]. One limitation to measuring fetal estrogen is the high estrogen production by the placenta during mammalian pregnancy. Placental estrogen production led to the hypothesis that estrogen secretion by the ovaries is negligible compared to its high placental production. In contrast to the well-established embryonic androgen action, a local paracrine and autocrine estrogen action has not been identified so far. The embryonic testis produces anti-Müllerian hormone (AMH) and testosterone. AMH causes regression of the Müllerian ducts and the onset of testicular descent. Testosterone causes the differentiation of the Wolffian ducts. In mice, *NR2F2* represses the development of the Wolffian ducts during female development (Figure 2). Little is known about the role of estrogens in the development of the female genitalia. Current models are still based on the assumption that the absence of testicular testosterone and AMH production is sufficient to produce female sex differentiation – underpinning the default or passive way of female development [22, 29].

### 2.3 The role of estrogens in sex development

One observation suggesting that estrogens influence the differentiation of the genitalia comes from the effect of the synthetic estrogen diethylstilbestrol (DES) that was given to pregnant women in order to prevent miscarriages in the 1970s. While the treatment did not show the desired effect, boys exposed to DES during embryonic development were born with an increased incidence of hypospadias (where the urethra opens on the underside of the penis or between the anus and the scrotum), indicating that estrogens at supraphysiological doses can inhibit male genital development. Female embryos exposed to DES showed an increased incidence of vaginal cancer later in life. Interestingly, experiments performed in mice in the 2000s showed an effect on both male and female genital development when exposed to intrauterine DES [30]. Another study investigated estrogen action by knocking out the *Esr1* in mice. Female *Esr1* knock-out mice developed a longer clitoris with cartilage, which is typical of the penis but not for the clitoris [31]. This indicates a suppressive role for estrogens in preventing male sex development and calls into question the default model of female sex development.

Steroid hormones act through steroid hormone receptors, namely the estrogen receptors 1 (ESR1) and 2 (ESR2) that are activated by estrogens and the androgen receptor that is activated by androgens. Much of the current knowledge about the role of androgens in male sex development has been derived from studying complete androgen insensitivity syndrome (CAIS). Affected individuals possess male gonosomes and testosterone-producing gonads, yet develop a female phenotype with a blind-ending vagina, and no Müllerian structures (namely no uterus) or prostate due to the absence of a functional androgen receptor. This syndrome has been used as an explanatory model for the role of androgens in the development of the male inner and outer genitalia (Figure 2). It also led to the conclusion that the absence of androgen is sufficient for female sex differentiation. This conclusion, however, is not valid. Individuals with CAIS do produce estrogens and have functional estrogen receptors and are therefore unsuitable for studying estrogen action. In addition, the development of inner female organs cannot be studied in this model as male gonads are functional in AIS and produce AMH that impedes the Müllerian structures’ development. The only available model to study estrogen action in human sex development at the organism level is complete estrogen resistance, an extremely rare condition in humans. Due to its rarity, mutations in *ESR* genes and the underlying molecular mechanisms of estrogen insensitivity have not been thoroughly studied. Individuals with mutations in *ESR1* show a hypoplastic uterus and ovaran dysfunction (32). Loss of function mutations, in the human *ESR2* are associated with ovarian dysgenesis a hypoplastic uterus and hypoplastic labia majora in females. Interestingly, in men *ESR2* mutations lead to testicular dysgenesis, suggesting that this receptor is involved in gonadal development in both sexes [32]. *Esr1*-deficient female mice show a hypoplastic uterus and reduced fertility and an enlarged clitoris [29], indicating both an activating role in producing female sex organs and an inhibitory role in preventing male sex development.

Research on sex development in biology follows a model of causality, in which sex chromosomes are the basis of gonad formation which are the basis of steroid hormone production. This model is also based on assumptions of the two sexes, as illustrated by the idea of a master switch in male sex development. DSD contradicts this model and therefore gives space for a more complex model of sex where the concerted action of different not necessarily binary factors give rise to an individual sex (Figure 2).

## 3 Science is not a mirror of nature

It would be simplistic to believe that the explanation of human sex development created by biologists is a one-to-one reflection of natural processes. As feminist commentators have argued, the science of embryogenesis and development cannot be “a ‘mirror of nature’ but rather an interpretive grid through which people narrativise phenomena” [33]. “[C]omponents of our political, social, and moral struggles” are necessarily interwoven in the categories and concepts used in biology to understand and interpret empirical findings [34]. To deny this would underestimate the challenge biology faces regarding the complexity of multicellular processes. This is especially pertinent in explanations of molecular mechanisms of sex determination. Binary concepts, as Fausto-Sterling argues, led “researchers to ignore data which are better accounted for in approaches which accept the existence of intermediate states of sexuality” [35].

We highlight three strands of critical thought about the concept of sex genes. A first strand deals with the structures of causality: is causality uni-directional and top-down or are causative relations in sex development better thought of as bi-directional, context-related and part of (epi-)genetic networks? A second strand criticises dualistic assumptions in genetic explanations, which implicitly relate to a strictly binary sex or gender system. A third strand is the androcentric bias that has been diagnosed by feminist science studies in some of the seemingly neutral and objective genetic and biochemical analyses of sex development [36].

**Table 1:** Strands of critical thought about the concept of sex genes

	**Critique**	**Concern**
1	Genetic determinism	No awareness of contextual factors
	Genetic essentialism	Gene’s-eye view on embodiment
2	Dualism	Neglect of intermediates
3	Androcentrism	Naturalised social injustice

### 3.1 Causality: Genetic determinism and essentialism

The gene *SRY*, at the time of its identification in 1990, seemed to confirm a pre-existing model of how genetic sex determination works: one single “master” gene on the Y chromosome directs the development of the testes and thereby determines the male sex. However, even in the late 1990s this model was heavily challenged from two sides: firstly by empirical findings, which complicated the picture considerably, and secondly by conceptual considerations about how genes work in an organism. More genes seem to be involved, some of them non-Y chromosomal genes (such as *NR0B1, SOX9, DMRT1* and* WNT4*), which could override *SRY* to cause sex reversal. *SRY* did not offer a sufficient explanation of the phenotypes of human intersex subjects. Ken McElreavey et al. therefore suggested a “regulatory gene cascade”, in which many factors participate in pushing the balance of sex determination towards male or female [37]. This pluralistic model of determination would better explain the observed variations of intersex or DSD (differences of sex development) phenotypes. Sarah Richardson argues in her excellent review of this discussion that gender criticism played a considerable role in this transition away from genetic determinism and essentialism, and toward a contextual, process-oriented view of complex (epi-)genetic networks that emphasise the interacting factors involved in sexual development [38]. These theories are based on the idea that the role of some genes as genetic “switches” is not the cause but the *result* of complex interactions with other factors.

Genetic determinism, coupled with an essentialist view of the role of the genome in organismic development, has a long history going back to August Weismann’s late 19th century belief that the chromosomes contain a plan (“Architekturplan”) of the developing organism. Long before details could experimentally be elucidated, a general theoretical view promised an explanation of the role of the genome in the organism: the genome contains the information of a “genetic program” that enables the organism to develop the adult structure. Eminent biologists including Ernst Mayr, Jacques Monod and François Jacob successfully promoted this view in the early 1960s [39], because it solved the theoretical puzzle of how seemingly goal-directed developmental processes could be explained mechanistically. However, not least due to findings such as mRNA editing and alternative splicing indicating the contextual nature of genetic causality, a general uneasiness with genetic determinism and genetic essentialism arose in philosophy of biology.

“Systemic” explanations preserve the causal importance of genes while avoiding genetic determinism and essentialism. They have increasingly been recognised as more congenial to empirical findings [40, 41].

### 3.2 Dualism

Thinking in dualisms manifests itself in the division of autosomal and sex chromosomes, or in the distinction between X and Y chromosomes. While in public the X chromosomes are described as “motherly”, more sociable, conservative, stable [42], the Y chromosome is often seen as active, dominant, a “macho entity” [43]. These divisions often relate to a dichotomy of active and passive, since in Western culture masculinity is seen as an active agent and femininity is seen as passive. Dualism also assumes sex binarity, i. e. the view that there are only two sexes and no variants in between.

Dualism also resonates with the (now challenged) view that female sex development is a “default” pathway that occurs naturally without the effects of an (active) male-determining gene. In the view of biological femininity as absence, sex determination research has focused on questions of the genetics of male determination. As we argued in section 2, this view is inconsistent with a series of recent findings that show that the female developmental pathway is in many ways the result of “active” genetic and hormonal processes.

### 3.3 Androcentrism

The genetics of sex determination developed in a traditionally male-dominated scientific world. From there it has clearly inherited an androcentric bias, for instance by focusing on the Y chromosome and on testosterone and by looking for male “master” genes. The mechanisms of embryogenesis have been investigated with primary attention to the development of the male phenotype [44]. Testis formation was seen as the crucial sex-determining event, female sexual development as “default”. Researchers saw “the question of *sex determination* as identical to the question of genetics of *male testes determination*” [38]. As a consequence female sex development was under-researched and is today much less well understood than male sex development.

## 4 Outlook: where now?

As all this shows, concepts and theories of sex differences are based not only on facts but also on cultural and social factors that influence how research is done and how scientists make sense of its results. Changing cultural assumptions about gender and new models beyond the binary to describe biological phenomena will therefore play a key role for our understanding and also for the future of sex genes. There is no biology without history and no materiality or body without discourse – the entanglements and complexities of sex and gender, of cultural and biological dimensions, which is always the context in which scientific research is embedded, can never be “relegated to the footnotes” [33].

Chromosomal, genetic and hormonal determinations of sex difference provided the framework of argumentation for assuming a natural duality of biological sex excluding all naturally occurring variations of sex development. An androcentric scientific environment focused on the study of male sex development and perpetuated the notion of a default and passive route to female sex development. Far fewer genes involved in ovarian development have been identified than for testis development, with the consequence that many patients with ovarian dysgenesis remain without a genetic diagnosis. Knowledge of the role of estrogens in human sex development lags behind the well studied androgen action. Finally, the (epi)genetic factors leading to the differentiation of the inner and outer sex organs in sex development are still largely unknown.

Biotechnological developments such as CRISPR technologies are making sex chromosomes, the hard core of “sex itself”, [38], mutable and changeable. Epigenetic research shows that biological processes of gene expression and gene regulation interact with external, social, biographical and long-term factors – outside the body. Alongside technological and scientific development, social and cultural changes have fostered the recognition of diverse and multiple forms of sex and gender, which are not represented in the cultural matrix of a two-gender model. Will binary models be replaced by discrete concepts of multifactorial variables, similar to the brain mosaic approach [45]? And will studies of sex and gender medicine primarily pursue the goal of improving health and providing human rights-compliant medicine, rather than on finding the holy grail of the source of gender differences?

Sarah Richardson argues for a new approach to the study of sex differences, which she calls “sex contextualism” [46]. She understands sex contextualism as a framework for the operationalisation of “sex” in biomedical research, in contrast to essentialism, which conceptualises sex as a single component and ignoring the context of research. Sex contextualism does not ignore the ubiquity of sex variables in research materials, or overlook the different understandings and complexities of sex and gender difference, and it does not so easily fall into generalising arguments about ourselves.

Biosocial approaches simultaneously question the reductionisms of sociobiology and of cultural constructionism, and propose instead an integration of the biological with the social [47]. The approaches of “biocultural integration” as a merging of human sciences with molecular biology have been intensively discussed over the last 10 years [48, 49]. The impetus for these debates comes not only from social and feminist studies but also from the biological sciences, where the retreat from gene-centric explanations has been interpreted as a fundamentally new style of thinking in molecular biology [50, 51].

## References

[1] Laqueur T (1992). Making Sex: Body and Gender from the Greeks to Freud. Making Sex: Body and Gender from the Greeks to Freud.

[2] Freud S (1969). Die Weiblichkeit [1933]. In: Mitscherlich A (ed.) Sigmund Freud. Studienausgabe. Vorlesungen zur Einführung in die Psychoanalyse und Neue Folge. Band 1.

[3] Kammerer P (1920). Die Geschlechter. Geschlecht und Gesellschaft 10.

[4] Stoff H (1999). Vermännlichung und Verweiblichung. Wissenschaftliche und utopische Experimente im frühen 20. Jahrhundert. In: Pasero U, Braun F (eds.) Wahrnehmung und Herstellung von Geschlecht: Perceiving and Performing Gender.

[5] Steinach E (1920a). Verjüngung durch experimentelle Neubelebung der alternden Pubertätsdrüse. Archiv für die Entwicklungsmechanik der Organismen 46.

[6] Steinach E (1920b). Künstliche und natürliche Zwitterdrüsen und ihre analogen Wirkungen. Drei Mitteilungen. Archiv für die Entwicklungsmechanik der Organismen 46.

[7] Sengoopta C (1998). Glandular politics. Experimental biology, clinical medicine, and homosexual emancipation in fin-de-siècle central Europe. Isis 89(3).

[8] Steinach E, Lichtenstern R (1918). Umstimmung der Homosexualität durch Austausch der Pubertätsdrüsen. Münchener Medizinische Wochenschrift 65.

[9] Hirschfeld M (1914). Die Homosexualität des Mannes und des Weibes. Die Homosexualität des Mannes und des Weibes.

[10] Hirschfeld M (1926). Geschlechtskunde auf Grund dreißigjähriger Forschung und Erfahrung. Vol. I: Die körperseelischen Grundlagen.

[11] Oudshoorn N (1994). Beyond the Natural Body: An Archaeology of Sex Hormones. Beyond the Natural Body: An Archaeology of Sex Hormones.

[12] Butenandt A (1934). Neuere Erkenntnisse in der Untersuchung der Sexualhormone. Forschungen und Fortschritte 10.

[13] Lipschütz A (1918). Die Gestaltung der Geschlechtsmerkmale durch die Pubertätsdrüsen. Archiv für Entwicklungsmechanik der Organismen 44.

[14] Steinach E (1912). Willkürliche Umwandlung von Säugetiermännchen in Tiere mit ausgeprägt weiblichen Geschlechtscharakteren und weiblicher Psyche. Pflügers Archiv 144.

[15] Kronfeld A (1920). Kurze Übersicht über die Pubertätsdrüsen-Frage. Geschlecht und Gesellschaft 10.

[16] Berner O (1928). Maskulinisation durch Ovarialgeschwülste. In: Marcuse M (ed.) Verhandlungen des I. Internationalen Kongresses für Sexualforschung. Vol. II: Physiologie, Pathologie und Therapie.

[17] Stevens NM (1905). Studies in spermatogenesis. Studies in spermatogenesis.

[18] Bridges BC (1939). In Allen E, Danforth CH, Doisy EA. Sex and Internal Secretions. 2nd ed.

[19] Sturtevant AH (1945). A Gene in Drosophila Melanogaster That Transforms Females into Males. Genetics 30(3).

[20] Danon M, Sachs L (1957). Sex chromosomes and human sexual development. Lancet 273(6984).

[21] Hughes IA, Houk C, Ahmed SF, Lee PA (2006). Consensus statement on management of intersex disorders. J Pediatr Urol 2.

[22] Reyes AP, León NY, Frost ER, Harley VR (2023). Genetic control of typical and atypical sex development. Nature Reviews Urology.

[23] Sinclair AH, Berta P, Palmer MS, Hawkins JR, Griffiths BL, Smith MJ, Foster JW, Frischauf A-M, Lovell-Badge R, Goodfellow PN (1990). A gene from the human sex-determining region encodes a protein with homology to a conserved DNA-binding motif. Nature 346(6281).

[24] Elzaiat M, McElreavey K, Bashamboo A (2022). Genetics of 46,XY gonadal dysgenesis. Best Practice & Research. Clinical Endocrinology & Metabolism 36(1).

[25] Elzaiat M, Todeschini A-L, Caburet S, Veitia RA (2017). The genetic make-up of ovarian development and function: The focus on the transcription factor FOXL2. Clinical Genetics 91(2).

[26] Kousta E, Papathanasiou A, Skordis N (2010). Sex determination and disorders of sex development according to the revised nomenclature and classification in 46,XX individuals. Hormones 9(3).

[27] Meinel JA, Yumiceba V, Künstner A, Schultz K, Kruse N, Kaiser FJ, Holterhus P-M, Claviez A, Hiort O, Busch H, Spielmann M, Werner R (2023). Disruption of the topologically associated domain at Xp21.2 is related to 46,XY gonadal dysgenesis. Journal of Medical Genetics 60(5).

[28] George FW, Wilson JD (1978). Conversion of androgen to estrogen by the human fetal ovary. The Journal of Clinical Endocrinology and Metabolism 47(3).

[29] León NY, Reyes AP, Harley VR (2019). A clinical algorithm to diagnose differences of sex development. The Lancet. Diabetes & Endocrinology 7(7).

[30] Mahawong P, Sinclair A, Li Y, Schlomer B, Rodriguez E, Ferretti MM, Liu B, Baskin LS, Cunha GR (2014). Prenatal diethylstilbestrol induces malformation of the external genitalia of male and female mice and persistent second-generation developmental abnormalities of the external genitalia in two mouse strains. Differentiation 88(2–3).

[31] Yang JH, Menshenina J, Cunha GR, Place N, Baskin LS (2010). Morphology of mouse external genitalia: Implications for a role of estrogen in sexual dimorphism of the mouse genital tubercle. The Journal of Urology 184(4).

[32] Biason-Lauber A, Lang-Muritano M (2022). Estrogens: Two nuclear receptors, multiple possibilities. Molecular and Cellular Endocrinology 554.

[33] Cipolla C, Gupta K, Rubin DA, Willey A (2017). Queer Feminist Science Studies: An Introduction. In: Queer Feminist Science Studies: A Reader Seattle.

[34] Fausto-Sterling A (2020). Sexing the Body: Gender Politics and the Construction of Sexuality (Second Edition). Sexing the Body: Gender Politics and the Construction of Sexuality (Second Edition).

[35] Fausto-Sterling A (1989). Life in the XY-Choral. Women’s Studies International Forum 12(3).

[36] Anderson E (2020). Feminist Epistemology and Philosophy of Science, The Stanford Encyclopedia of Philosophy. (Spring 2020 Edition).

[37] McElreavey K, Vilain E, Abbas N, Herskowitz I, Fellous M (1993). A regulatory cascade hypothesis for mammalian sex determination: SRY represses a negative regulator of male development. Proceedings of the National Academy of Sciences of the United States of America 90(8).

[38] Richardson SS (2013). Sex Itself. The Search for Male and Female in the Human Genome. Sex Itself. The Search for Male and Female in the Human Genome.

[39] Kay LE (2000). Who Wrote the Book of Life? A History of the Genetic Code. Who Wrote the Book of Life? A History of the Genetic Code.

[40] Neumann-Held EM, Rehmann-Sutter C (2006). Genes in Development: Re-Reading the Molecular Paradigm. Genes in Development: Re-Reading the Molecular Paradigm.

[41] Love AC (2008). Explaining the Ontogeny of Form: Philosophical Issues. In: Sarkar S, Plutynski A (eds.) A Companion to the Philosophy of Biology.

[42] Angier N For motherly X chromosome, Gender is Only the Beginning. For motherly X chromosome, Gender is Only the Beginning.

[43] Marshall Graves JA (2000). Human Y Chromosome, Sex Determination, and Spermatogenesis- a Feminist View. Biology of Reproduction 63(3).

[44] Page DC, Mosher R, Simpson EM, Fisher EM, Mardon G, Pollack J, McGillivray B, Brown LG (1987). The Sex-Determining Region of the Human Y Chromosome Encodes a Finger Protein. Cell 51(6).

[45] Joel D (2021). Beyond the Binary: Rethinking Sex and the Brain. Neuroscience and Biobehavioral Reviews 122.

[46] Richardson SS (2022). Sex Contextualism. Philosophy, Theory, and Practice in Biology 14(2).

[47] Ingold T, Palsson G (2013). Biosocial Becomings: Integrating Social and Biological Anthropology. Biosocial Becomings: Integrating Social and Biological Anthropology.

[48] Keller EF (2016). The Postgenomic Genome. In: Richardson S, Stevens H (eds.) Postgenomics. Perspectives on Biology After the Genome.

[49] Meloni M (2014). How Biology Became Social, and What It Means for Social Theory. The Sociological Review 62(3).

[50] McDade TW, Harris KM (2022). From society to cells and back again: New opportunities for discovery at the biosocial interface. Discover Social Science and Health 2(4).

[51] Zhao F, Yao H (2019). A tale of two tracts: history, current advances, and future directions of research on sexual differentiation of reproductive tracts. Biology of Reproduction 101(3).

